# How models can make a difference for a sustainable future of the building industry

**DOI:** 10.1617/s11527-018-1287-8

**Published:** 2018-11-27

**Authors:** Klaas van Breugel

**Affiliations:** Delft University of Technology, Delft, The Netherlands

**Keywords:** Modelling, Sustainability, Ageing, Infrastructure, Quality, Self-healing

## Abstract

Models play a vital role in science and technology and in the evolution of modern societies. They are used for describing processes and mechanisms, experimental observations, analyses and predictions. In spite of the fact that models are a reduction of reality, the achievements of modern societies are impressive and would have been inconceivable without the role of models. These achievements can be qualified with one term: growth. The impressive growth in past decades, however, now appears to be unsustainable. In this contribution the above-mentioned dilemma is dealt with. First the evolution and use of models is discussed from a more general sustainability perspective. In the second part of the paper the application of models for sustainable solutions in the field of cementitious materials is emphasized.

## Introduction

Throughout history people have been confronted with hazards and threats. The notion of threat also appears in one of the most rudimentary definitions of architecture: Architecture is the art and science of providing *shelter* for people. People need shelter to protect themselves. Threat could come from the climate, the environment, hostile tribes, etc. Shelter could be provided by houses, protective walls and dams, city walls or watch towers. In his masterpiece ‘Chance und Risiko der Gegenwart’ the philosopher Staudinger [1] points out that, apart from providing physical shelter, people have also tried to get into *control* of the circumstances to which they were exposed. For that purpose they turned to shamans and witchdoctors. These persons were supposed to be able to make contact with the invisible world behind and beyond the everyday reality. Through their contacts with the invisible world shamans and witchdoctors were supposed to be able to manipulate the circumstances people had to cope with, to change their circumstances for better ones or to protect themselves against unavoidable threat.

In his book Staudinger describes how the concept of two worlds, i.e. the real world around us and the invisible world behind it, still applies today. Like in the past, also today people want to be in control. For that purpose, however, they do no longer expect answers from shamans and witchdoctors. Galilei’s (1564–1642) [2] often quoted statement that ‘the book of nature is written in mathematics’ is an expression of the evolution of thinking about reality. Newton’s [3] ‘Mathematical Principles of Natural Philosophy (1684)’ was a next step on the way to find ‘the formula’ according to which the reality functions. No longer the gods and ghosts determine how things go, but everything follows the laws of nature. Beyond the visible world, we could say, there is an invisible world of models, the ‘scientific replica’ of the real world. The real world was no longer seen as holy an untouchable, but a reality that is accessible for science and research. Step by step the world became demythologised.

**Fig. 1:**
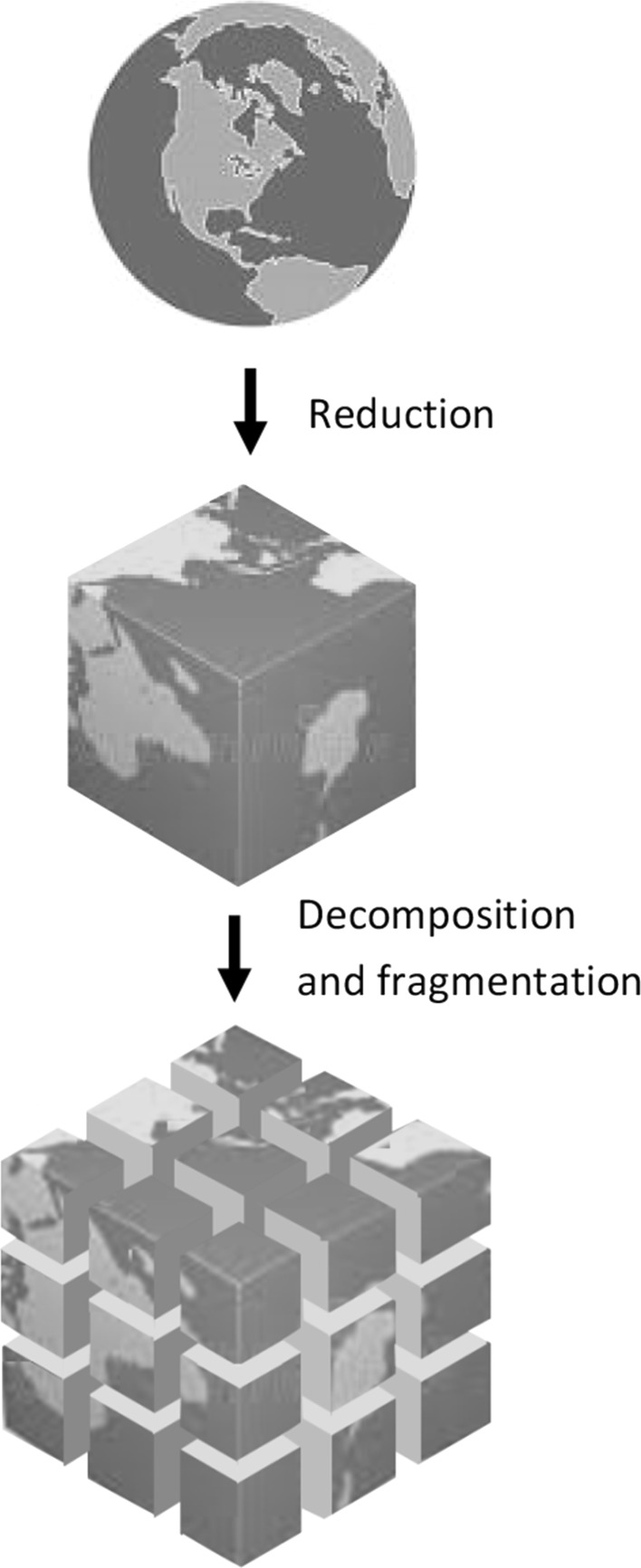
From real world to the reductionist (multidisciplinary) scientific approach

This process of demythologization has been described and judged by, among others, Von Weizsäcker [4]. He draws attention to the *reductionist* approach in science, particularly in scientific modelling. The reductionist character of science and scientific modelling means that however sophisticated a model might be, it is still a simplification of reality. Schematically is shown in Fig. 1. The figure schematically illustrates how the real world (top of the figure) is reduced from a perfect sphere to an ‘imperfect’ one, i.e. a cube, and this cube is finally sub-divided in individual domains, separated by interfaces. Through reduction and decomposition scientists try to disclose the secrets encountered in their own domain and to understand the ‘grammar’ according to which the real world behaves. Once this grammar is understood, scientist and engineers are able to create their own alternative world. This process has, in the end, resulted in our modern built environment. In spite of being no more than an approximation of reality, models and the use of them became the new vehicle to ‘manipulate’ reality and design a new technology-based society.

When we look from a distance to the forgoing we could say that in the past, in the absence of knowledge and understanding of natural laws, people tried to get grip on reality by turning to a secret world behind the visible world. Today people want the get grip on reality by breaking it down into elementary units. These elementary units are used to ‘engineer’ a world that meets the needs and wishes of a modern society.

**Fig. 2:**
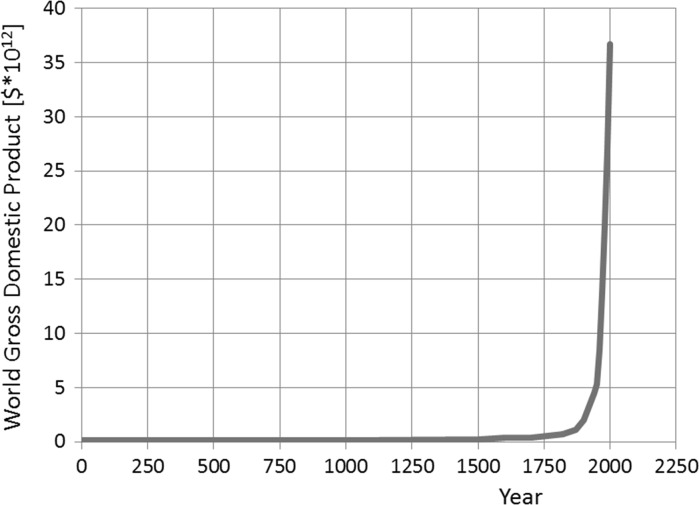
Evolution world Gross Domestic Product (after Roser [5] and The Maddison Project [6])

The achievements resulting from progress in science and technology can be illustrated by the dramatic increase in the world’s gross domestic product, shown in Fig. 2. This figure shows an unprecedented growth. Today there is no indication that this growth will stop. There is still a demand for further growth, particularly in countries with growing economies, like China and India. Meanwhile negative side effects of this unprecedented growth have revealed themselves and are now intensively discussed. From these discussions we have learned at least two things. Firstly, our increased knowledge and understanding of nature has enabled us to create modern industrialized societies, suggesting that we are pretty much in control. Secondly, the unprecedented growth in the past two centuries has been achieved at the cost of the environment and is now judged unsustainable, suggesting that we are *not* in control. It seems fair to say that science and technology have been developed and exploited *in the service of growth*, without having paid sufficient attention to the environment. In so far models and modelling have contributed to growth, the currently encountered sustainability issues reveal and illustrate the consequences of the reductionist nature of the models that were used. The challenging question is now whether models, that have contributed so much to growth in the past, can similarly contribute to the development of sustainable construction in the future.

## The infrastructure stock and our responsibility

### Value of the infrastructure stock

The growth in the world GDP, presented in Fig. 2, goes along with a similar growth of the global infrastructure stock. The value of the world’s infrastructure has been estimated between US$ 51 and 125 trillion [7]. Still there is a huge demand for further growth. In 2013 Dobbs et al. [8] estimated the need for future investments in the infrastructure at about US$ 57 trillion to enable and ensure the prognosticated economic growth in the period 2013–2030. This means an investment of US$ 3.2 trillion per year in the indicated period. This figure includes infrastructure for transport (roads, (air)ports, rail), water, telecommunication and power plants.

The investment of US$ 57 trillion in the period 2013–2030 is the budget needed for *growth*. In this period also huge budgets are needed for renovation, repair and replacement of *existing* infrastructure. In this context it is good the remember that the design lifetime of civil structures is 50–80 years. This means that structures built between the fifties and seventies of the past century have now reached the end of their service life. Some structures are still in good condition, while others have to be replaced by new ones. If we estimate the value of the world’s infrastructure stock at US$ 90 trillion and assuming an average lifetime of 75 years, the costs for replacement are US$ 1.2 trillion per year. This amount has to be spent on top of the aforementioned amount US$ 3.2 trillion per year needed for *growth*. For both replacement of existing structures and growth a budget is needed in the coming 18 years of US$ 4.4 trillion per year. The figures are summarized in Table 1. A substantial share of the investments are needed for concrete structures.

**Table 1:** Estimated worldwide investment in infrastructure in the period 2013–2030

Category	Estimated budget	
	US$/year	%
Replacement existing infrastructure	1.2 trillion	27
Growth infrastructure stock	3.2 trillion	73

### Raw materials

**Fig. 3:**
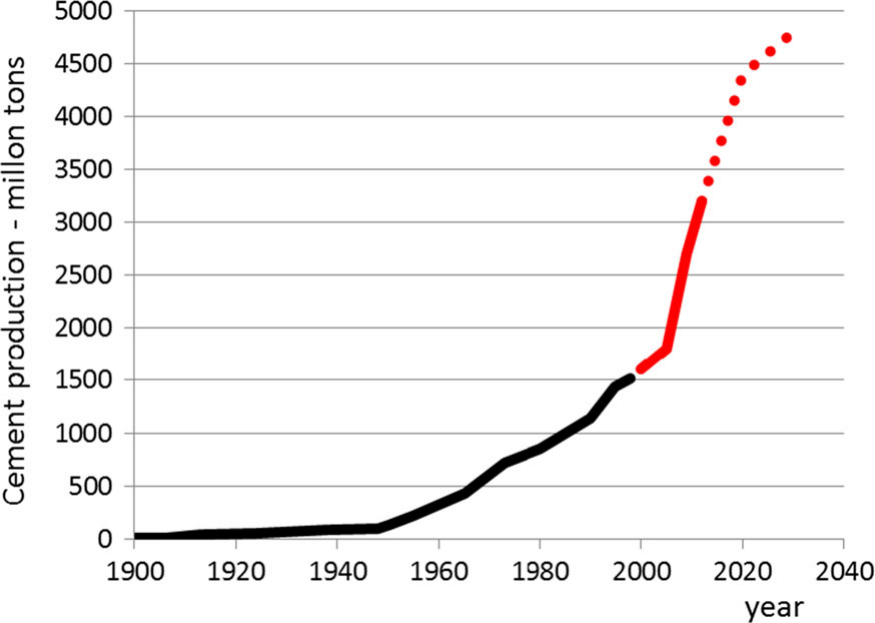
Past (until 2000) and prognosticated (2000–2030) growth of cement consumption (Based on data from Aitcin [12] and Statista [13])

Growth implies an increasing demand for building materials. Figure 3 shows how the cement production is expected to increase from about 1.5 billion tons in 2000 to 3.5 billion tons in 2015 and 4.4 billion tons in 2030. Even though the quantities of raw materials worldwide are huge, there can be a *local* shortage of them. Sand, gravel and rock are present in abundant quantities, but have not always the required properties for producing good quality concrete. Ignoring quality criteria, or unawareness of the properties of local materials, can result in the use of inappropriate materials and large-scale premature failure of concrete structures, as exemplified by the pyrrhotite drama in Quebec and Connecticut [9, 10]. A local shortage may stem from a real absence of raw materials in a certain district, but can also be the result of local restrictions to exploit available resources. Local shortages of raw materials will lead to long-distance transport of bulk materials. If mitigation of the footprint of the construction industry is our goal, long-distance transport of bulk goods should be minimized as much as possible. This implies a recommendation of the use of local resources for producing alternative building materials [11].

### Environmental impact of growth: CO_2_ emissions

The production of building materials and the building process itself go along with emissions. CO_2_ emissions associated with the production of steel and concrete are estimated at 3.25 and 3.44 billion tons per year, respectively [7]. This is about 20% of the worldwide CO_2_ emission per year [14]. Cleaner production of building materials would be welcome. However, in past decades both the steel and cement industry have already made big steps towards cleaner production processes and further dramatic reductions are hardly conceivable. Moreover, the effect of reducing the CO_2_ emissions per ton produced steel or concrete will soon be overtaken by the global growth in the use of steel and concrete, particularly in countries with a rapidly growing economy [7].

A substantial share of the global CO_2_ emissions originate from construction-related transport [15]. Vrijhoef [16] estimated that 30–40% of the all transport is related to construction activities, representing some 40% of vehicle emissions. It is conceivable that a substantial reduction of CO_2_ emissions can be accomplished easier by reducing construction-related transport than by reducing the emissions associated with the production of steel and concrete.

## Modelling in the service of sustainable construction

### From growth to sustainable development

The growth curve in Fig. 2 indicates that the unprecedented growth of the world GDP started in the second half of the nineteenth century, i.e. the period of the industrial revolution. That is also the time that concrete was invented and steel production started to increase dramatically. The large-scale availability of these building materials were crucial for building an extensive infrastructure. This infrastructure, on its turn, stimulated further growth. Both science and technology have played an important role in this process of growth, while models and modelling were the vehicles to make science and technology operational in the practice. As mentioned earlier this in this paper, in the past science and technology have been used and further developed *in the service of growth*. In the forgoing sections, however, we have also pointed to the harmful implications of continuous growth. Metaphorically speaking we can say that growth should result in maturity. Growth *beyond* maturity results in an unhealthy situation. Call it obesity. It’s a disease. It’s unsustainable. The intriguing question is now whether models and modelling can also be used to cure this disease. It seems that we don’t have time anymore to discuss this question at length. The urgency of the environmental problems tells us that we have to act. It’s time to use models and modelling *in service of sustainable construction*! Using models and modelling in the service of sustainability does not exclude the option of growth, but growth should be *sustainable* growth, aiming at maturity.

### Coping with ageing

**Fig. 4:**
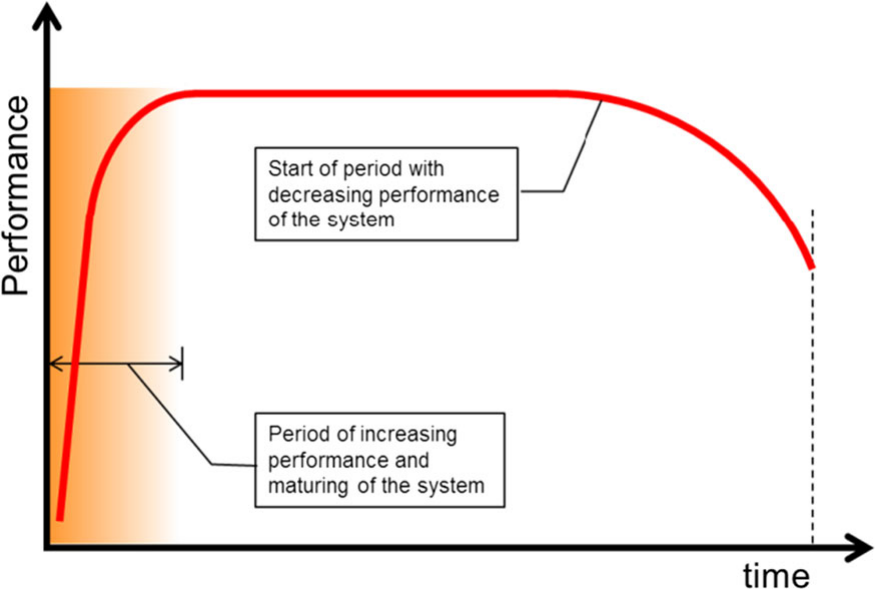
Evolution of the performance of ageing materials, structures and systems

Although sustainable construction involves more than reducing CO_2_ emissions, there is no doubt that mitigation of these emissions is one of the key-goals of sustainable development. Reductions of CO_2_ emissions, as well as investments needed to accomplish these reductions, should be considered in relation to the total lifetime of a structure. From the sustainability point of view a long service life is favourable, at least if not realised against extremely high costs for repair and maintenance. The typical performance curve of any material, structure or system is shown in Fig. 4. In the initial phase, i.e. the construction phase, the structure gradually matures until the required level of performance is reached. During the structure’s lifetime prevailing performance criteria have to be met at reasonable maintenance and repair costs. Both the service life and the maintenance and repair costs strongly depend on the *initial quality* of the structure [17]. Good initial quality will result in lower maintenance and repair costs during the structure’s lifetime and hence contributes to sustainable construction. In view of fact-based asset management and future recycling, the initial quality of a structure, including data on materials used and execution, should be documented in a birth certificate [18, 19].

However good the initial quality might be, during the service life *ageing* of the structure will start and will lead to a decrease of performance [20]. Particularly in high quality structures these ageing processes will develop very slow. Yet, these slow ageing processes determine the service life of the structure. For existing building materials ageing processes are still poorly understood. This holds even more for new, alternative building materials, promoted because of their claimed environment friendliness. The extremely low rate of ageing processes constitute a great challenge for material scientists and modellers. Here fundamental models can make a difference!

In the following a few examples are presented of modelling activities in view of design of low CO_2_ binders, and simulation of performance phenomena like micro-cracking and self-healing of cement-based materials.

## Modelling topics in view of sustainable construction

### Hydration and microstructure development: Blended cement pastes

Almost a century ago a start was made with developing models for simulation of hydration processes in cement-based materials [21, 22]. The first models were, of course, relatively simple and focused on hydration of individual cement components, e.g. C_3_S and C_3_A. Another simplification was that hydrating particles were assumed to react independently from each other. No allowance was made for any effect of physical interaction and microstructure formation on the rate of reaction. With the increase of computation power it became possible to deal with more complex systems and the interaction between reaction rate and microstructure development could be considered (integrated kinetic models [23]). The progress in modelling has meanwhile been described in several review papers [24, 25]. With many of the proposed models the rate of hydration can be predicted sufficiently accurate to serve engineering purposes, for example in curing control systems.

Besides the rate of hydration the evolution of the microstructure and the pore structure are inherent parts of the output of these simulation models. The microstructure is the basis for the description of the evolution of the materials properties like strength and stiffness, whereas the pore structure determines the transport properties.

**Fig. 5:**
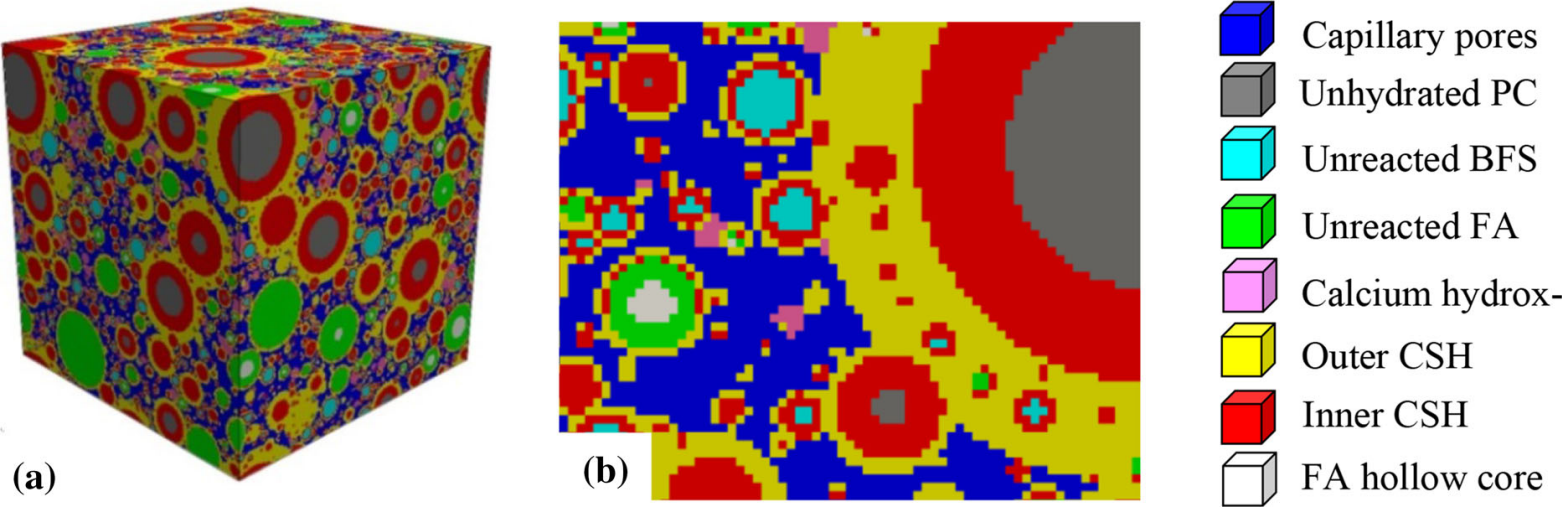
The digitalization of the simulated 3D microstructure of blended cement paste: **a** Simulated microstructure visualized in 3D, **b** Digitalized microstructure visualized in 2D (*w*/*b* = 0.4, 60% PC, 20% BFS, 20% FA, see the properties of PC, BFS and FA in Section 5.5) [26]

Although these simulation models are still quite young and need further refinement, there is a demand already for upgrades of them in order to deal with more complex binders with a low CO_2_-profiles, i.e. binary and ternary systems. Figure 5 shows a result of a 3D numerical simulation of a ternary system, calculated with an extended version of the HYMOSTRUC program [26]. The mixture contains Portland cement, fly ash and blast furnace slag. From the generated virtual microstructure strength parameters can be inferred, i.e. contact areas between individual particles.

**Fig. 6:**
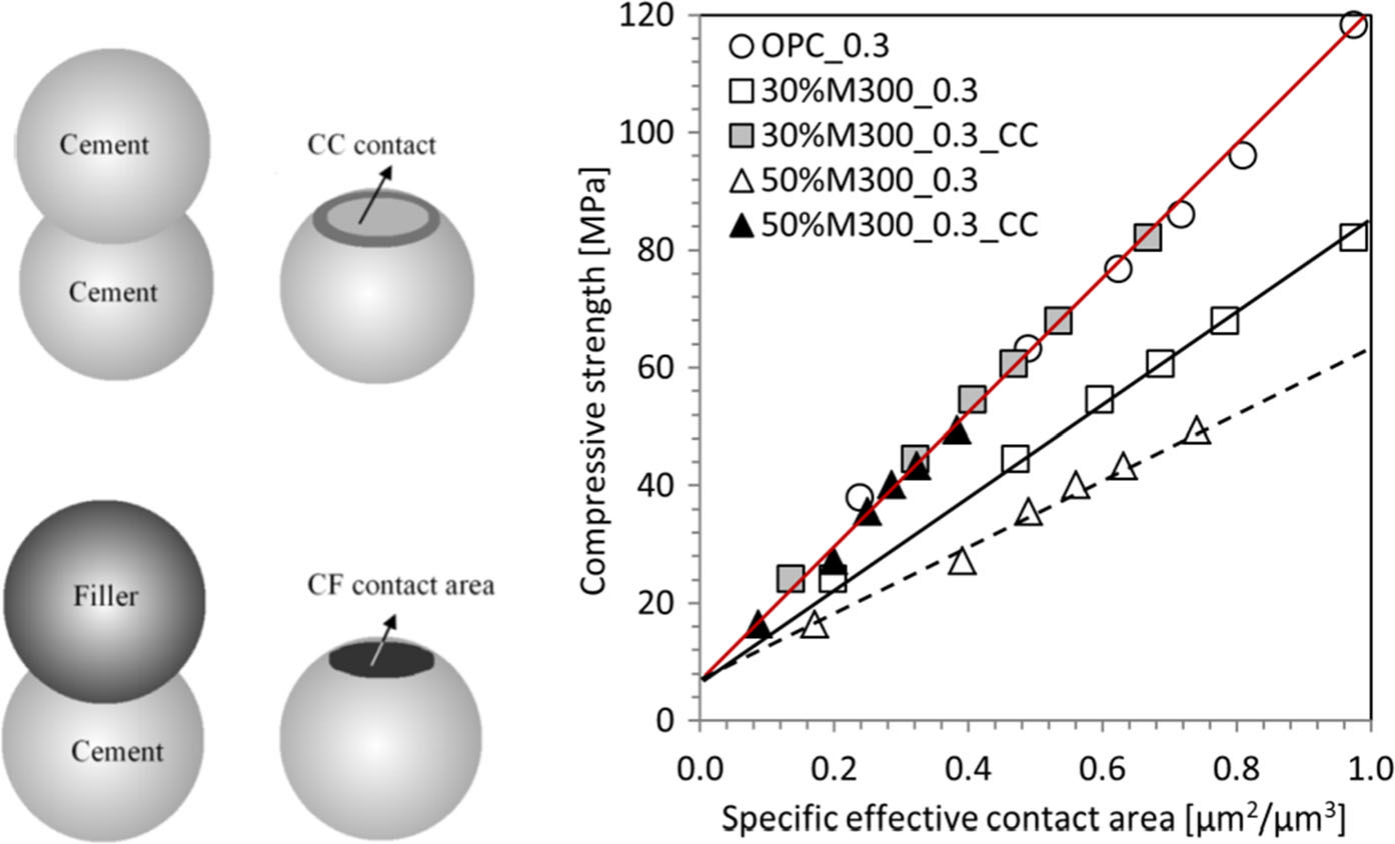
Measured compressive strength vs calculated interparticle contact area of cement pastes and pastes blended with micronized sand. Replacement percentage 30 and 50%. *w*/*b* = 0.3 [27]

Ouyang [27] used virtual microstructures for analysing and optimizing binder systems blended with either micronized sand or limestone powder. A linear correlation was found between the calculated interparticle contact area and the compressive strength. In his analyses he differentiated between the strength of the contact area between cement particles (CC-surface) and between cement and filler particles (CF-surface). Figure 6 shows a typical result for pastes in which 30 and 50% of the cement was replaced by micronized sand. It was found that the contact area between cement particles and micronized sand particles hardly contributed to the compressive strength. All the mixtures showed a unique liner relationship of the contact area between the cement particles (CC-surface) with the compressive strength.

Currently used simulation models have shown to be quite successful for simulating hydration and microstructure development. Temperature predictions and simulations of short-term mechanical properties can be carried out quite accurately. But there are still tedious modelling issues left. First, the pore structure of the small pores is difficult the model accurately. The connectivity of small pores is different for different types of cement. Zhang [28] showed that particularly in case of non-saturated concrete the characteristics of the small pores are decisive for the transport properties. For currently used simulation models it is tough to describe these small pores accurately. Second, models for hydration and microstructure development don’t consider the development of *hydration*-*induced stresses* and *microcracking*. Microcracking may already occur in hydrating cement *paste*, and is even more likely to happen in hardening *concrete*. These microcracks will affect the long-term performance of the concrete. Further modelling can help to understand the driving forces behind these stresses and the resulting fracture processes.

### Microcracking

**Fig. 7:**
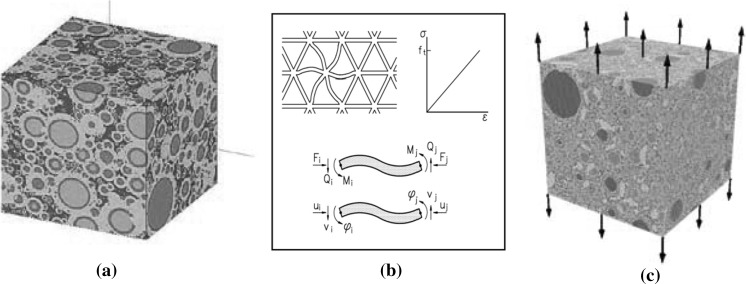
Virtual 3D microstructure (left) as basis for generating a lattice mesh (right) for Delft Lattice Model analyses. Sample 100 × 100 × 100 µm^3^, loaded in tension [29]

The fracture process in a virtual cement paste microstructure has been analysed by, among others, Qian [29] and Schlangen et al. [30]. For simulation of the fracture process these authors used the Delft Lattice Model (Fig. 7b, [31]). The starting point of the stress analysis was a virtual microstructure (Fig. 7a). The cement grains are connected by ‘beams’, which form a three dimensional network. The mechanical properties of the lattice system are a function of the size of the contact area between (partly) hydrated cement particles. The virtual microstructure is then loaded in tension in a strain-controlled virtual test (Fig. 7c). If the stress in one of the beams exceeds the assigned strength, the beam will be “removed” from the lattice system and a new equilibrium is then found by iteration.

**Fig. 8:**
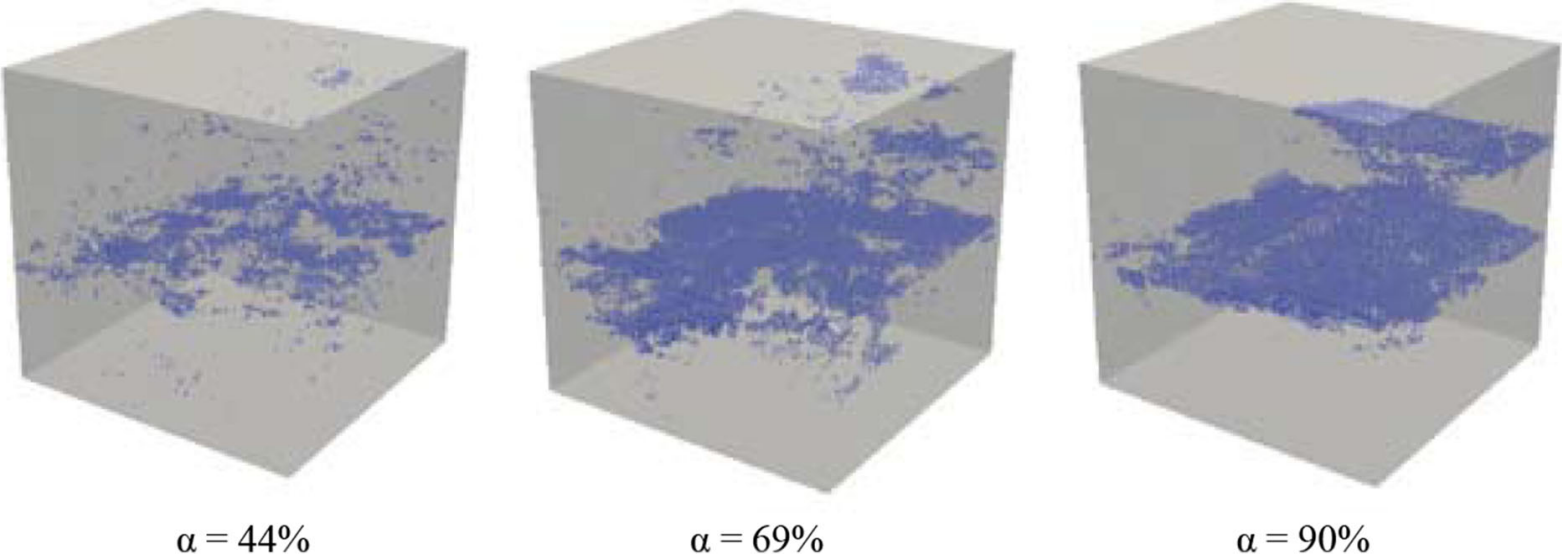
Cracking pattern in virtual microstructure of cement paste. w/c = 0.4. Degree of hydration is 44, 69 and 90%, respectively. Samples 100 × 100 × 100 µm^3^ [30]

Figure 8 shows crack patterns of a virtual cement paste specimen, w/c 0.4, loaded in tension (strain controlled) at a degree of hydration of 44, 69 and 90%, respectively. In the specimen with a low degree of hydration the cracks are spread throughout the volume of the specimen. When pastes with a higher degree of hydration are loaded, cracks are more localised. In the latter case fewer microcracks are formed and the specimen performs more brittle. Similar results were found by Dao et al. [32] in experimental studies of tensile properties of early age concrete.

### Self-healing of cracks

**Fig. 9:**
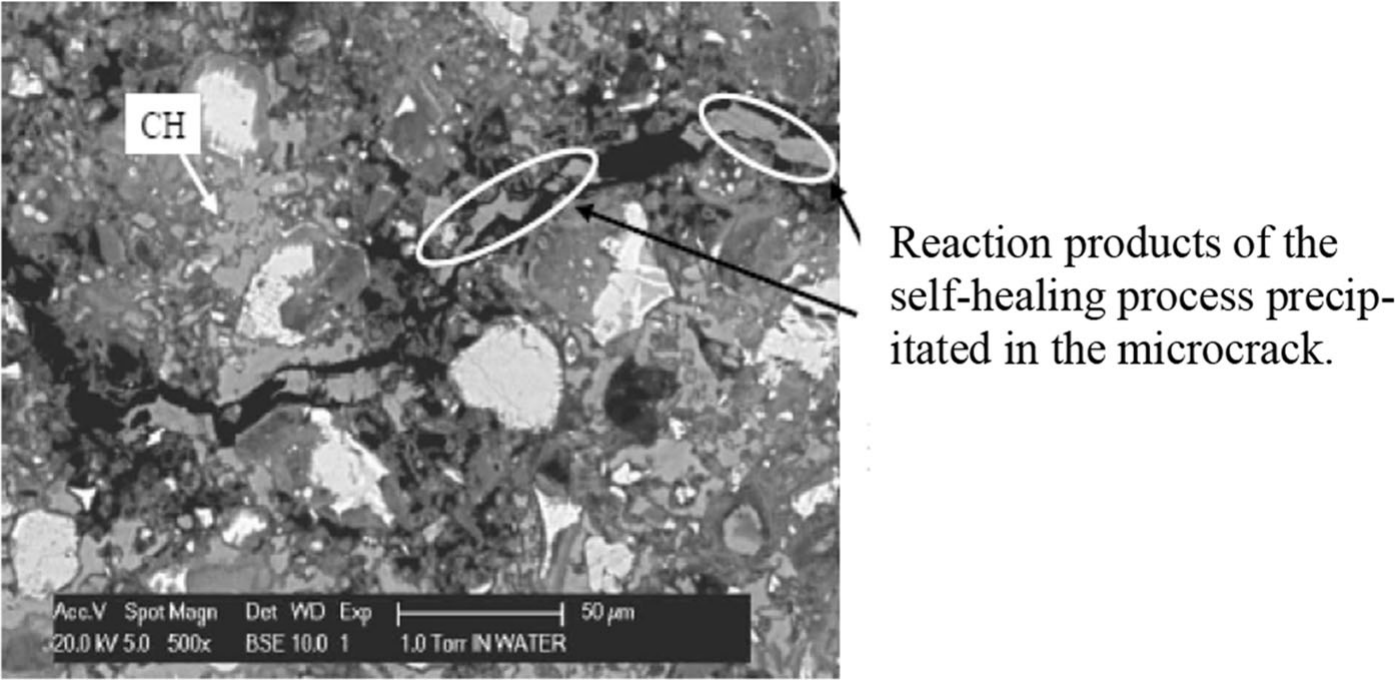
BSE images of a micro-crack in Portland cement paste after 200 h curing in water. Self-healing tests started at an age of the cement paste of 28 days [33]

**Fig. 10:**
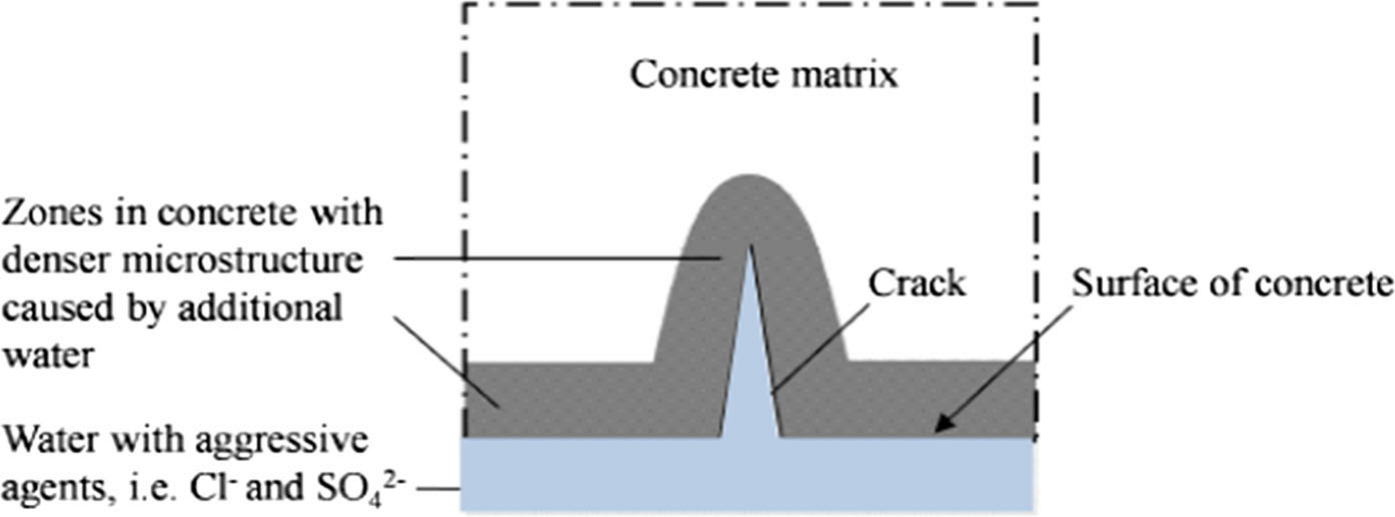
Densification of matrix adjacent to a crack surface after external water supply [33]

Micro-cracks are considered to increase to proneness of concrete to deterioration. Micro-cracks, however, may also heal because of the inherent self-healing capacity of concrete. With the aim to simulate the self-healing mechanism in cracked concrete Huang [33] studied the precipitation of reaction products in a micro-crack. Figure 9 shows an example of a BSE-image of an investigated micro-crack. NMR studies of moisture movements showed that, once a micro-crack is formed and water penetrates into the crack, the relatively dry paste will immediately absorb some water. This water is then available for further hydration of still unhydrated cement cores in the bulk paste adjacent to the crack. As a result the zone around the micro-crack will become denser. Schematically this is shown in Fig. 10. In fact we see here ‘pure’ self-healing of the material due to densification of the matrix around the crack.

Besides local densification of the paste adjacent to the crack, the crack itself is gradually filled with reaction products. Simulations showed that the healing mechanism will benefit from the presence of relatively large cement grains, i.e. coarsely ground cement. This might explain why older structures, made with coarse cement, are less prone to ageing and perform better than modern ones built with finer and more rapid cements [34].

### Volume changes at early ages

In the early stage of hydration there are continuous changes of the microstructure, the water content and the distribution of water in the pore system. These changes are associated with volume changes, either swelling or shrinkage. At macro level we only observe the net result of these swelling and shrinkage mechanisms. In low water-cement ratio mixtures the volume changes are dominated by the shrinkage mechanism. The resulting deformation of those low *w*/*b* ratio mixtures is known as autogenous shrinkage. Autogenous shrinkage is supposed to be strongly related to the self-desiccation process. In sealed concrete the self-desiccation process will stop when the hydration process stops. Experiments reveal, however, that autogenous shrinkage continues even after the hydration process has virtually ceased [35–37]. These ongoing deformations suggest that creep is involved here. These creep deformations would follow the elastic deformations, the latter supposed to be caused by the capillary forces associated with the self-desiccation process.

The foregoing shows that at least two issues complicate the interpretation of measured deformations of hardening cement paste. First, the (partly) simultaneously acting swelling and shrinkage mechanisms. Second, a possible contribution of creep. It is not easy to identify the contribution of creep in a continuously changing material. By quantitative modelling of individual mechanisms the potential contribution of these mechanisms to the resulting deformation can be evaluated. Is it believed that models are indispensable for clarifying this complex issue.

## Sustainability construction: the broader picture

### Circularity

Today circularity is a key-issue in many fields, also in the building industry. In fact, circularity in the building industry is not new. Waste materials have been (re)used for centuries already [38]. Although it is not realistic to assume that 100% circularity is possible, it is at least a concept that generates the badly needed awareness of the environmental impact of the construction industry and forces us to think about alternative concepts.

**Fig. 11:**
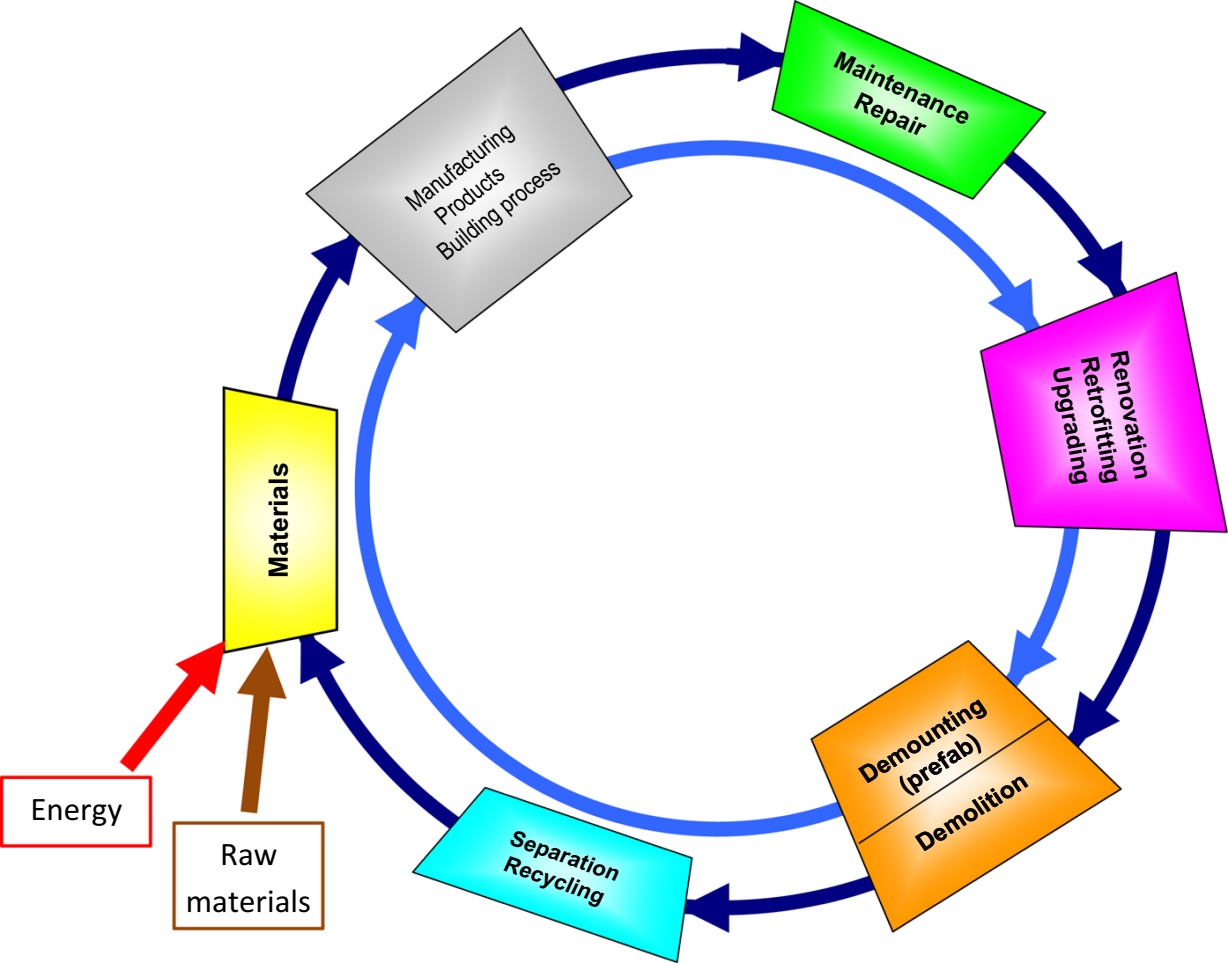
Building cycle. Outer circle: monolithic design concept. Inner circle: demountable design concept. Consumption of energy and raw materials to be reduced as much as possible (after [39])

Figure 11 shows a schematic of the building cycle with subsequent stages of the life cycle of a structure. It starts with the design and production of building materials. In the next phase construction elements and/or complete structures are produced. The outer circle of Fig. 11 represents the so-called *monolithic design concept*, whereas the inner circle represents the *demountable design concept*. After having passed the stage of renovation, retrofitting and up-grading and having reached the end of their service life, structures built according to the monolithic design concept will be demolished. In a circularity concept the demolition waste will be crushed and reused for producing new building materials. Structures built according to the demountable concept will provide structural components for reuse in new buildings directly.

The inner circle (demountable design) will require less energy for a complete loop compared to the outer circle (monolithic design) and is, therefore, preferable from the sustainability point of view. Of course, the reuse of construction elements cannot go on forever. Ageing of the material and degradation processes dictate the number of life cycles of structural components. Finally demolition and crushing of structural components has to be envisaged.

It is conceivable that for *replacement* of existing structures recycling of old structures is an option. Old structures can serve as *donor buildings* for new buildings. Yet, a situation that one single obsolete structure can provide *all* the required materials for the structure by which the obsolete one will be replaced, will be exceptional. It is conceivable, however, that collecting materials from a large number of obsolete and demolished buildings may provide an amount of ‘second life’ material large enough for realizing new buildings (urban mining). Quality control of this second life materials and logistics are crucial factors for making this concept economically and technically attractive.

The circularity concept is considered to be an instrument for mitigating the environmental footprint of the construction industry. In a situation of *growth*, however, it is less obvious that a circularity concept shown schematically in Fig. 11 can offer much solace. Growth is foreseen in countries where not many buildings are available yet for providing second life material. In most situations where growth has to be realized there is almost nothing to recycle yet! The data in Table 1 clearly shows that—globally—the expected investments for growth are more than two times the investments needed for replacement of old structures. Since recycling can only be marginal in a situation of growth, circularity is not expected to contribute much to short-term mitigation of the environmental footprint on the global scale. Hence, in a situation of *growth* sustainable solutions should focus preferably on using as little material as possible, minimizing transport, using the lowest amount of energy as possible, minimizing the maintenance and operation costs and realizing structures with appropriate initial quality in order to ensure the required service life.

The forgoing does not mean that in situations of growth the circularity concept has no relevance. In the left bottom corner of the scheme of Fig. 11 it is indicated that the building cycle always needs some external input, i.e. energy and raw materials. In a ‘perfect’ circular economy these inputs are minimal, whereas in a situation of growth the demand for energy and raw materials will by far dominant the input from, for example, demolition waste. For realising sustainable growth it is recommended to design materials and structures in such a way that, once the end of service life of a structure is reached, the materials or structural components of obsolete structures easily fit in a low-energy circular concept. Models can be of great help to optimize this process.

### Serious games for making the difference

The circularity concept, illustrated with Fig. 11, is a challenging concept for modelling, numerical simulations and optimization. It must be emphasized, however, that the schematic of Fig. 11 primarily focuses on the materials circle and does not show the high multi-disciplinarity of the whole building process. Optimization of the building process, with the aim to accomplish sustainability goals, requires active participation of *all* stakeholders in the construction chain. It is particularly at the level of multidisciplinary collaboration that models can be instrumental for making the difference. The type of modelling that is appropriate to deal with multidisciplinarity is that of serious gaming [40, 41]. A serious game provides a platform for all stakeholders, i.e. materials scientists, technologists, designers, architects, craftsmen, owners, politicians, economists, land-use planners, teachers etc., to collaborate with a common interest and common goal to improve the quality of their joint efforts and of their final product. Their common overall goal is, in short, to contribute to a sustainable society. With reference to the bottom-cube of Fig. 1, we could say that collaboration of individual stakeholders presupposes that they cross the interfaces, or gaps, between their own domains and work together. In a serious game interdisciplinary collaboration can be simulated and improved. The effect of decisions made by any of the stakeholders on the quality of their product and on the environmental impact in terms of CO_2_ emissions, transport costs, structural safety, depletion of materials etc., can be simulated. Knowing that poor communication is one of the key-reasons for failure and inferior quality, serious games should focus on improving communication between disciplines. Serious games can bring the whole design and building process closer to individual craftsmen, which enhances their commitment to the construction process and gives them the possibility to judge the relevance of their contribution to the quality of the final product. All these characteristics, features and goals of serious games make them a perfect strategic tool for enhancing the performance of the constructions industry.

## Benefits of using models and modelling

Developing models is a time consuming activity. Consequently, it is also an expensive activity. Yet, science is inconceivable without modelling. Moreover, models are also *beneficial*, both in research and engineering. Some of these benefits are obvious, for example if there is no alternative for using models. Service life predictions, for example, are not possible without using models. Models can support complex experimental studies of the performance of a material or a structure under combined loading. Judging the benefits of modelling in industrial manufacturing processes is less easy. In this respect it is worthwhile to take notice of an extensive study of Boucher [42] on cost saving strategies in the manufacturing industry. In her study she also investigated the benefits of using simulation models. The study involved 157 companies in a variety of industries. Based on the outcome of the study three classes were defined: Best-in-Class (Top 20%), Average (Middle 50%) and Laggard (Bottom 30%). It was found that simulation tools and technologies were “extremely powerful and provided great benefits to a company, enabling to make better design decisions”. The Best-in-Class companies were more than 3 times more likely to take a systematic approach to simulation. “Because the Best-in-Class companies integrated the use of simulation throughout the entire design process, they got more out of it. Engineers were able to make better informed decisions on an ongoing basis throughout the design process, which led to fewer engineering change orders later on in the addition to lower costs and higher quality products”. These are important lessons and find support from individual industrial companies [43]. Particularly for a complex manufacturing industry, like the building industry, the potential of numerical simulations can hardly be overestimated.

## Conclusions

Modelling has always been an important activity in science. Models are used to test hypotheses, to simulate processes and to predict performance. Moreover, their use has been instrumental in establishing the modern built environment, involving huge amounts of raw materials and energy.

In spite of the long tradition of modelling and impressive achievements by using models, models are still a reduction of reality. Attempts to (re)shape or create a new reality with the help of reductionist models will sooner or later reveal the shortcomings of these models. This is not problematic as long as we are aware of possible shortcomings and prepared to cope with them once they reveal themselves. In fact this holds at all conceivable levels of modelling. Awareness of shortcomings is a precondition for starting a search for improvements. In this contribution two directions have been discussed where improvements of models are required and conceivable. Models for hydration and microstructure development have to be extended in view of the use of blended systems and for detailed predictions of the pore structure and long-term performance. Ongoing studies aiming at connecting microscale models to nanoscale and ab-initio models are promising and inevitable to force breakthroughs. These breakthroughs can be the development of new high performance materials, but also improved understanding of ageing phenomena and how to mitigate the effects of these phenomena. At the other end of the modelling spectrum the development of serious games was mentioned. Serious games are believed to have the potential to improve the performance of the building process in terms of product quality and mitigation of the environmental impact. Typical for serious gaming of construction activities is that clear sustainability goals can be defined, which are guiding for all stakeholders active in the construction chain. In the past these goals were dominated by growth. Sustainable development, however, requires a different perspective. In the future science and technology, as well as models and modelling, should no longer be developed and used in the service of growth but in de service of sustainable development. Serious games can facilitate this change of perspective. This will certainly take time. But don’t despair. It has been remarked that developing a more or less accurate model of the globe took more than 2000 years [44]. Hopefully models and serious games for sustainable construction will take less time.
